# Association of *toll-like receptor 3* polymorphism rs3775291 with age-related macular degeneration: a systematic review and meta-analysis

**DOI:** 10.1038/srep19718

**Published:** 2016-01-22

**Authors:** Li Ma, Fang Yao Tang, Wai Kit Chu, Alvin L. Young, Marten E. Brelen, Chi Pui Pang, Li Jia Chen

**Affiliations:** 1 Department of Ophthalmology and Visual Sciences, The Chinese University of Hong Kong, Hong Kong, China; 2 Department of Ophthalmology and Visual Sciences, Prince of Wales hospital, Hong Kong, China

## Abstract

Association of a polymorphism rs3775291 in the *toll-like receptor 3* (*TLR3*) gene with age-related macular degeneration (AMD) had been investigated intensively, with variable results across studies. Here we conducted a meta-analysis to verify the effect of rs3775291 on AMD. We searched for genetic association studies published in PubMed, EMBASE and Web of Science from start dates to March 10, 2015. Totally 235 reports were retrieved and 9 studies were included for meta-analysis, involving 7400 cases and 13579 controls. Summary odds ratios (ORs) with 95% confidence intervals (CIs) for alleles and genotypes were estimated. *TLR3* rs3775291 was associated with both geographic atrophy (GA) and neovascular AMD (nAMD), with marginally significant pooled-P values. Stratification analysis by ethnicity indicated that rs3775291 was associated with all forms of AMD, GA and nAMD only in Caucasians (OR = 0.87, 0.78 and 0.77, respectively, for the TT genotype) but not in East Asians. However, the associations could not withstand Bonferroni correction. This meta-analysis has thus revealed suggestive evidence for *TLR3* rs3775291 as an associated marker for AMD in Caucasians but not in Asians. This SNP may have only a small effect on AMD susceptibility. Further studies in larger samples are warranted to confirm its role.

Age-related macular degeneration (AMD) is a common degenerative disease among the elderly population, leading to distorted central vision in the early stage and severe visual loss in the advanced stage. The prevalence of early AMD is about 8.01% and advanced AMD 0.37%^1^. Advanced AMD has two subtypes: geography atrophy (GA, or advanced dry AMD), characterized by extensive atrophy of the retinal pigment epithelium (RPE) and the overlying photoreceptors, and neovascular AMD (nAMD, also known as exudative or wet AMD), characterized by choroidal neovascularization (CNV).

AMD is a complex disease with multiple genetic and environmental factors. A recent multi-cohort genome-wide association study (GWAS) has identified 19 AMD loci, accounting for approximately 15–65% of total genetic contribution to AMD^2^. The *age-related maculopathy susceptibility 2* (*ARMS2*) and *complement factor H* (*CFH*) are the major associated genes for AMD. However, the *ARMS2* risk allele seemed to be more associated with nAMD, whereas the *CFH* risk allele was preferentially associated with GA^2^, suggesting differences in the genetic profiles between GA and nAMD. There are also differences in their responses to treatment. While there is no effective therapy for GA, the anti-vascular endothelial growth factor (VEGF) therapy has improved the vision of many patients with nAMD^3^.

It has been reported that the inflammatory cascades are involved in the pathogenesis of AMD^4^. One of the key proteins in the inflammatory response is toll-like receptor 3 (TLR3)^5^. It recognizes viral double-stranded RNA (dsRNA) and induces apoptosis in infected cells^6^,^7^. A common single-nucleotide polymorphism (SNP) rs3775291 in the *TLR3* gene reduced the dsRNA-induced cell death of the RPE cells *in vitro* and *in vivo* and had shown potential clinical significance^8^. The SNP rs3775291, a C to T transition at nucleotide position 1234 (c.C1234T), leads to a leucine to phenylalanine substitution at amino acid 412 (p.Leu412Phe). This SNP did not affect the expression level of TLR3^9^, but it was found to reduce the binding capacity of TLR3 to dsRNA and protected against GA^7^. Moreover, TLR3 was upregulated during CNV formation in the association analysis between TLR3 and human CNV membranes^10^. These findings provided evidence for the association of *TLR3* rs3775291 with different subtypes of AMD.

*TLR3* rs3775291 was first found in Americans of European descent to protect against GA but not affect CNV or early AMD^8^. But subsequent studies in other populations suggested that rs3775291 was not associated with GA^11^,^12^. In a meta-analysis involving 3 reports, the T allele of rs3775291 had a protective effect for GA (odds ratio [OR] = 0.75, 95% confidence interval [CI] = 0.62–0.93)^7^. Recently, a lot more new data on rs3775291 have been reported in GA and other AMD subtypes^13^,^14^,^15^,^16^, revealing inconsistent association profiles in different study cohorts. Therefore, we conducted a meta-analysis to evaluate the role of *TLR3* SNPs, including rs3775291 and others, on different subtypes of AMD.

## Results

### Characteristics of eligible studies

**Figure 1:**
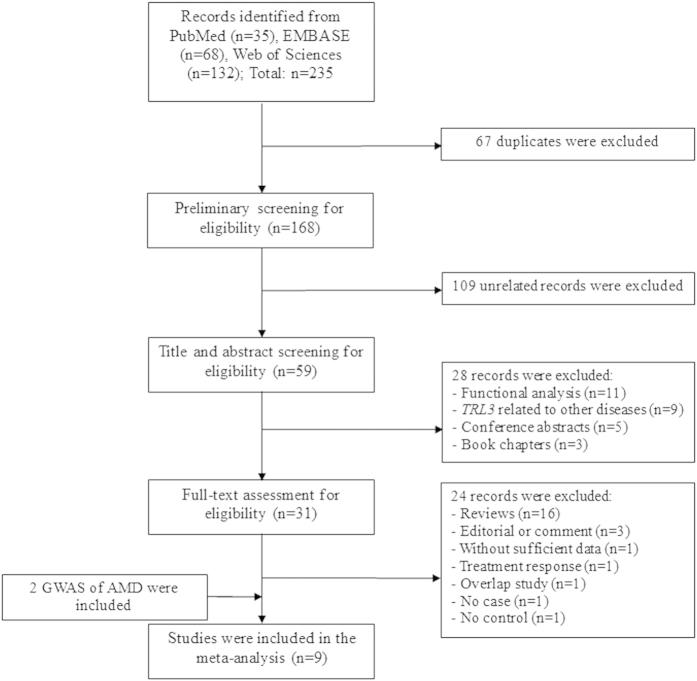
PRISMA flow diagram for inclusion of the studies investigating the association between *TLR3* rs3775291 and age-related macular degeneration. GWAS: genome-wide association study.

Figure 1 shows the flow of study inclusion in this meta-analysis. In brief, based on our search strategy, 235 records were identified in the initial search. After removing duplicates and studying the contents, we obtained 31 studies for further assessment. After full text review, 24 of them were excluded. We also manually searched the texts and supplementary materials of all reported GWAS of AMD. Consequently, 4 more relevant studies were identified^17^,^18^,^19^,^20^. However, samples in two studies^17^,^18^ were included in a later study with larger sample size^19^. Therefore, only the latest article was included for meta-analysis^19^. Finally, a total of 9 studies with 25 case-control cohorts were included, involving 7400 cases and 13579 controls^8^,^11^,^12^,^13^,^14^,^15^,^16^,^19^,^20^.

The main characteristics of the included studies are shown in Supplementary Table S1. Of the 25 cohorts, 21 were Caucasian, 3 Chinese and 1 Indian. The genotype distribution in controls followed Hardy-Weinberg equilibrium (HWE, P > 0.05) in every study except one^15^. Totally 12 SNPs have been tested in AMD. Three SNPs, rs3775291, rs5743303 and rs5743312, have been reported in at least two studies and are thus eligible for meta-analysis. The other 9 SNPs (rs4986790, rs5743305, rs3775290, rs11721827, rs11730143, rs11732384, rs13126816, rs10025405 and rs6830345) were investigated in single studies, in which they were reported to have no significant association with AMD^8^,^13^,^14^.

### Meta-analysis of *TLR3* rs3775291 in all forms of AMD

**Figure 2:**
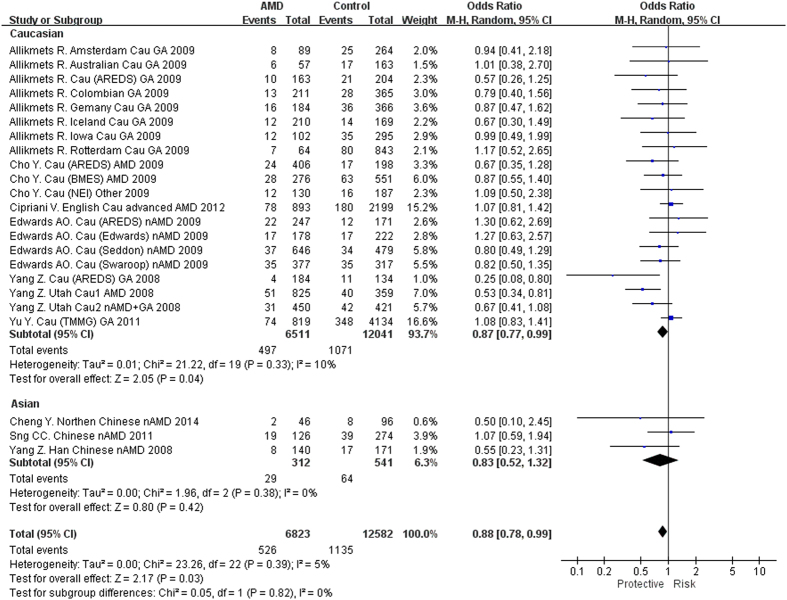
Forest plot of *TLR3* rs3775291(T) in all forms of AMD in the recessive model. Squares indicate study-specific odds ratios (ORs). The size of the box is proportional to the weight of the study. Horizontal lines indicate 95% confidence intervals (CI). A diamond indicates the summary OR with its corresponding 95% CI. AMD: age-related macular degeneration.

All these 25 case-control cohorts have been investigated for the association between *TLR3* rs3775291 and all forms of AMD (Supplementary Table S1). Of note, in the study of Cho *et al*. an overall association was evaluated in a subset of sample selected from 3 studies (age-related eye disease study [AREDS], National Eye Institute [NEI] and Blue Mountains Eye Study [BMES])^13^, therefore, the data of this sample subset was excluded from the meta-analysis. Accordingly, 24 cohorts from 9 studies were meta-analyzed. The pooled results indicated that rs3775291 was associated with all forms of AMD in the recessive model (P = 0.03, OR = 0.88, 95% CI: 0.79–0.99; Fig. 2), but not in other models (Table 1). In the stratification analysis by ethnicity, rs3775291 showed an association with all AMD in the recessive model in Caucasians (P = 0.04, OR = 0.87, 95% CI: 0.77-0.99; Fig. 2), but the P value did not withstand the Bonferroni correction. In Chinese, no suggestive association was detected in any genetic models.

### Meta-analysis of *TLR3* rs3775291 in GA

**Figure 3:**
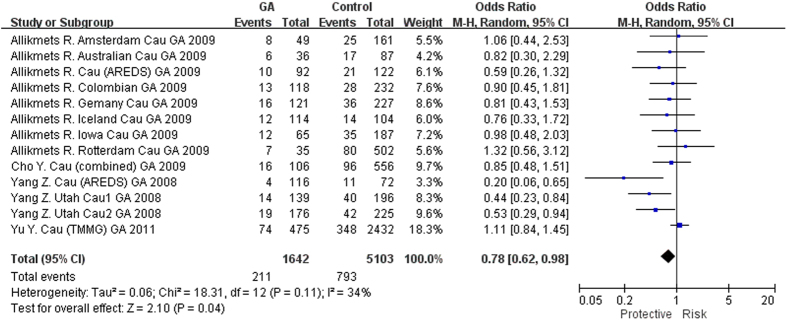
Forest plot of *TLR3* rs3775291(T) in GA in the homozygous model. Squares indicate study-specific odds ratios (ORs). The size of the box is proportional to the weight of the study. Horizontal lines indicate 95% confidence intervals (CI). A diamond indicates the summary OR with its corresponding 95% CI. GA: geographic atrophy.

Genotype data of rs3775291 was available from 6 studies with 19 cohorts for GA^8^,^11^,^12^,^13^,^15^,^19^. However, only the latest study was included^19^ from 2 reports with overlapping subjects^12^,^19^ and 1 report with combined data^13^. Finally, 5 studies^8^,^11^,^13^,^15^,^19^ with 14 case-control cohorts (13 Caucasian cohorts and 1 Indian cohort) were included in the meta-analysis, involving 2797 GA cases and 8714 controls (Table 2). The T allele showed a protective effect in the homozygous model with marginal significance (P = 0.04, OR = 0.78, 95% CI: 0.62-0.98; Fig. 3). Although there was no significant association in the other models, the ORs were toward the same trend (Table 2). There was no study on rs3775291 and GA in East Asians.

### Meta-analysis of *TLR3* rs3775291 in neovascular AMD

**Figure 4:**
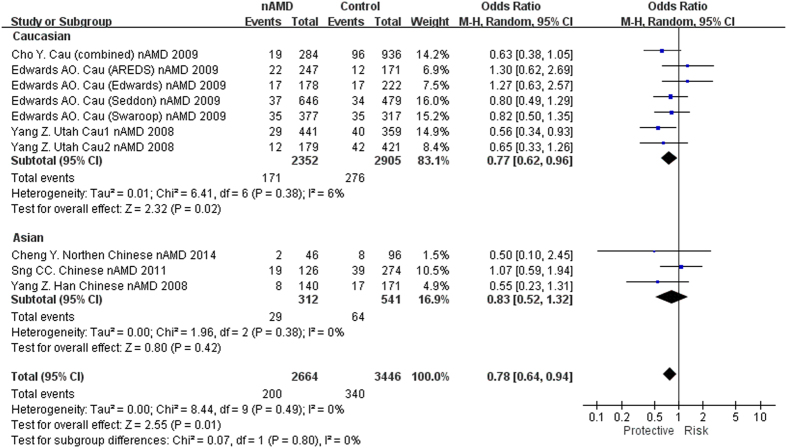
Forest plot of *TLR3* rs3775291(T) in nAMD in the recessive model. Squares indicate study-specific odds ratios (ORs). The size of the box is proportional to the weight of the study. Horizontal lines indicate 95% confidence intervals (CI). A diamond indicates the summary OR with its corresponding 95% CI. nAMD: neovascular age-related macular degeneration.

*TLR3* rs3775291 was reported in 6 studies with totally 11 cohorts^8^,^12^,^13^,^14^,^15^,^16^, including 2746 nAMD cases and 3507 controls (Table 3). Meta-analysis showed that this SNP was associated with nAMD in the recessive model (P = 0.01, OR = 0.78, 95% CI: 0.64–0.94; Fig. 4), but not in other models (Table 3). Subgroup analysis by ethnicity showed that rs3775291(TT) had a protective effect for nAMD in the recessive model in Caucasians (P = 0.02, OR = 0.77, 95% CI: 0.62–0.96; Fig. 4). However, the P values did not withstand the Bonferroni correction. In East Asians, no significant association was detected.

### Meta-analysis of *TLR3* rs5743303 and rs5743312 in AMD

Two studies investigated the association of rs5743303 with AMD in a total of 1695 cases and 1181 controls. Rs5743312 was tested by two studies in 1691 cases and 1181 controls. However, no significant association was detected for these 2 SNPs with AMD in any genetic models in this meta-analysis (data not shown).

### Sensitivity analysis and publication bias analysis

In the sensitivity analysis, the ORs for any genetic models were not substantively changed after removing the study deviated from HWE^15^. The significant associations were unchanged after removing any of the 9 studies, except the one by Yang *et al*.^8^. Moreover, neither the Egger’s test nor the Begg’s test identified any obvious evidence of publication bias (P > 0.1, Tables 1, 2, 3). In addition, the funnel plots did not seem to be asymmetric (Supplementary Figure S1). Thus, the results of this meta-analysis are relatively stable and unlikely to be affected by publication bias.

## Discussion

In this systematic review and meta-analysis, we have summarized the reported associations of *TLR3* rs3775291 with AMD. We found that this SNP was associated with combined AMD, GA and nAMD in the homozygous and/or recessive models, with the TT genotype showing a protective effect. In the subgroup analysis by ethnicity, rs3775291 showed a suggestive association with combined AMD (OR = 0.87, recessive model), GA (OR = 0.78, homozygous model) and nAMD (OR = 0.77, recessive model) in Caucasians, but not in East Asians. However, the associations could not withstand Bonferroni correction. Therefore, current data in the literature provides only suggestive evidence to support the role of *TLR3* rs3775291 in AMD susceptibility. Further studies in larger cohorts are needed.

The *TLR3* gene is located on chromosome 4q35, encoding 904 amino acids. The TLR3 polypeptide contains two characteristic Toll motifs: one is an extracellular leucine rich repeat domain and the other a cytoplasmic interleukin-1 receptor like region^21^. TLR3 expression was high in nAMD and in RPE cells in AMD donor patients, indicating involvement of TLR3 in the pathogenesis of nAMD^10^. Previous studies found that siRNAs targeting vascular endothelial growth factor-A or its receptor could suppress CNV via TLR3^22^,^23^. However, sequence- and target-independent siRNAs were also found to suppress CNV^24^. The choroidal endothelial cells from people expressing the TLR3 coding variant 412PhePhe (rs3775291) were found to be refractory to extracellular siRNA-induced cytotoxicity^24^. The variant p.Leu412Phe is located in the coding region near the site of glycosylation (Asn413), constitutes the ectodomain of TLR3 receptor and plays an important role in domain dimerization^25^,^26^,^27^. The dimerized ectodomain may provide a scaffold for RNA binding^24^. Although short RNAs may bind with TLR3, they need lengths of at least 21nt to activate TLR3^24^. Interestingly, these RNAs induced, rather than suppressed, AMD by activating TLR3 and triggering caspase-3-mediated apoptosis in RPE in mice^28^. These anti-CNV and AMD induction roles of TLR3 suggest its importance in GA. Ongoing efforts are to find out the sources of RNAs that bind to TLR3 to induce AMD.

The associations of *TLR3* polymorphisms and their roles in the pathogenesis of AMD have not been well defined as reflected by inconsistent results among different populations. In the study by Yang *et al*., rs3775291(T) conferred a protective effect against GA (P = 1.24 × 10^−7^) but not CNV or early AMD in Caucasians^8^. However, Edwards *et al*. reported no evidence of protection of rs3775291 in GA, with an opposite effect size (P = 0.75, OR = 1.05, 95% CI: 0.91–1.21)^12^. Although other studies reported rs3775291 was not associated with either GA or nAMD in Caucasian or Asian patients, the ORs showed a similar trend to the study of Yang *et al*., with the exception of three cohorts^11^,^12^,^16^,^19^ (Figs 2,3 and 4). So far the largest sample size for rs3775291 was found in a GWAS involving 819 AMD and 4134 controls, in which no association was detected (T allele, OR = 1.01, P = 0.82)^19^. Another GWAS involving 893 cases and 2199 controls also showed no significant association of rs3775291 with AMD (T allele, OR = 1.04, 95% CI: 0.92–1.18, P = 0.49)^20^. In GWAS only SNPs reaching a predefined threshold will be genotyped in replication cohorts, some SNPs might have been missed. Indeed, the statistical powers (<1%) for the two GWAS^19^,^20^ are insufficient to detect a significant association of rs3775291, assuming an OR of 0.9 and a predefine P threshold of 10^−5^. Therefore, results from multiple cohorts should be combined, e.g., by a meta-analysis, to validate the initial signals identified by candidate gene analyses. As summarized in our meta-analysis, suggestive genotypic (homozygous and recessive) associations were found in rs3775291 for all AMD, GA and nAMD, with the TT genotype showing a protective effect. Of note, leave-one-out sensitivity analysis indicated these significant associations were of no substantive change after removing any individual study (P < 0.05), with one exception^8^. This suggests that the study of Yang *et al*. might have played a predominant role in the current meta-analysis. However, as shown in the forest plots, the odds ratios of the TT genotype were towards the same direction in a majority of study cohorts, suggesting a same trend of effect conferred by the SNP. Nevertheless, the pooled-P values were at borderline, making it difficult to conclude on the association between *TLR3* and AMD. Further replication studies in more study cohorts with large sample sizes are needed. In addition, *TLR3* rs3775291 conferred protection against all AMD, GA and nAMD in Caucasians but not in Asians, indicating population-specific effect of rs3775291 in Caucasians. Moreover, in a previous study, we meta-analyzed the associations with PCV and found rs3775291 not associated with PCV (P = 0.09, OR = 1.25)^29^. *TLR3* activation may thus be implicated in the development of AMD rather than PCV.

The current meta-analysis is an overview of published genetic reports on *TLR3* rs3775291 in AMD, however, our study revealed several limitations. First, our findings were based on individual unadjusted assessment. Therefore, a more precise analysis should be adjusted for potentially confounding factors if individual data was available. Second, the meta-analysis in Asians, i.e., Chinese and Indians, was performed on a small sample size, involving only 312 cases and 541 controls from 3 studies. There was insufficient power to detect significant association with AMD (OR: 0.77–0.90 in our meta-analysis). Thus, the association of *TLR3* rs3775291 with AMD should be elucidated in larger Asian cohorts. Third, although genetic and environmental risk factors are considered as sharing a role to increase the risk of AMD, the included studies provide no gene-gene and gene-environmental interactions. Finally, no replication of the association of rs3775291 with early AMD has been reported since the study of Yang *et al*.^8^.

In summary, this systematic review and meta-analysis revealed that data in the current literature only provided suggestive evidence to support an association of *TLR3* rs3775291 with AMD in Caucasians but not East Asians, indicating ethnic diversities in the association profiles of *TLR3* in AMD. Since *TLR3* rs3775291 may confer only a small effect in AMD genetic susceptibility, larger cohorts should be recruited for further replication and validation.

## Methods

We used the Preferred Reporting Items for Systematic Review and Meta-Analysis statement (PRIMSA) for this systematic review and meta-analysis.

### Identification and selection of studies

Literature search was independently performed by two investigators (L.M. and F.Y.T.) for genetic studies on *TLR3* in the PubMed, EMBASE and Web of Science databases. All related studies reported up to March 10, 2015 were retrieved without language restriction. We used the following key words: (“toll-like receptor 3” or “toll like receptor 3” or “TLR3”) and (“age-related macular degeneration” or “AMD” or “ARMD” or “age-related macular disease” or “age-related maculopathy” or “ARM”). Furthermore, we also reviewed the reference lists of all retrieved studies to identify eligible studies. To maximize the usable data we also searched in all reported GWAS of AMD including their supplementary materials. Details of search strategy were shown in Supplementary Note.

### Inclusion and exclusion criteria

Eligible studies must meet the following criteria: (1) case-control studies; (2) exploration of association between *TLR3* and AMD; (3) raw data of allele or genotype counts available; (4) AMD was clearly defined by complete ophthalmic examinations, including fundus fluorescein angiography; (5) using elderly subjects without any other major eye diseases as controls; (6) for reports published by the same study group, the latest study or the one with the largest sample size was included for meta-analysis to avoid double counting. Reviews, case reports, conference reports, treatment response studies, editorial comments and reports without sufficient data were excluded.

### Data extraction

Two reviewers (L.M. and F.Y.T.) independently extracted the following information from each eligible study: name of first author, publication year, ethnicity, study design, diagnostic criteria of AMD, number of cases and controls, disease subtype, gender composition, mean age, allele and genotype distribution in the case and control groups, and Hardy-Weinberg equilibrium (HWE) test results in controls. Any discrepancies were resolved by thorough discussion with another reviewer (L.J.C.).

### Statistical analysis

We calculated the HWE with genotypes in the control group using Stata (version 12.0, StataCorp, College Station, TX, USA), if HWE was not given in the original study. Meta-analysis for each polymorphism was assessed if it had been reported in ≥2 studies. The association was performed using 5 genetic models, including allelic, dominant, recessive, heterozygous and homozygous models. The strength of association was estimated by calculating the summary ORs and 95% CIs. With respect to heterogeneity, the *I*^*2*^ statistics was used to estimate the degree of heterogeneity among the studies. Summary ORs and 95% CIs were calculated only by the random-effect model to yield more conservative CIs^30^,^31^. Sensitivity analysis was also conducted to examine the potential effect after removing the study diviated from HWE and each of the included studies one at a time^32^,^33^. All meta-analyses were conducted using the software Review Manager (RevMan, version 5.2, The Cochrane Collaboration, Copenhagen, Denmark). Also, publication bias among individual studies was calculated with the Egger’s test and Begg’s test by Stata^34^, where a P value of less than 0.1 was considered statistically significant. In association analysis, a pooled-P value of less than 0.05 was considered as suggestive evidence for a genetic association. Since we evaluated five genetic models, we applied the Bonferroni correction for multiple testing; thus a P value of less than 0.005 was required to conclude a statistically significant association.

## Additional Information

**How to cite this article**: Ma, L. *et al*. Association of *toll-like receptor 3* polymorphism rs3775291 with age-related macular degeneration: a systematic review and meta-analysis. *Sci. Rep*. **6**, 19718; doi: 10.1038/srep19718 (2016).

## Supplementary Material

Supplementary Information

## Figures and Tables

**Table 1:** Meta-analysis of *TLR3* rs3775291 in age-related macular degeneration.

Genetic model	No. of studies	No. of cohorts	Pooled sample size (AMD/control)	Meta-analysis of association			Publication bias (P)			
				OR (95% CI)	P	*I*^*2*^ (%)	Egger's Test	Begg's test		
T vs. C	9	24	6935/12643	0.97 (0.90–1.05)	0.47	48	0.59	0.62		
TT vs. CC	8	23	6823/12582	0.87 (0.75–1.01)	0.07	29	0.16	0.44		
TC vs. CC	9	24	6935/12643	1.03 (0.93–1.14)	0.54	45	0.94	0.82		
TT+TC vs. CC	9	24	6935/12643	1.00 (0.91–1.11)	0.96	50	0.77	0.79		
TT vs. TC+CC	8	23	6823/12582	0.88 (0.78–0.99)	**0.03**	5	0.068	0.44		

AMD: age-related macular degeneration; CI: confidence interval; OR: odds ratio; *TLR3*: *Toll-like receptor 3*.

**Table 2:** Meta-analysis of *TLR3* rs3775291 in geographic atrophy.

Genetic model	No. of studies	No. of cohorts	Pooled sample size	Meta-analysis of association			Publication bias (P)			
			(GA/control)	OR (95% CI)	P	*I*^*2*^ (%)	Egger's Test	Begg's test		
T vs. C	5	14	2797/8714	0.90 (0.80–1.02)	0.09	58	0.49	0.50		
TT vs. CC	4	13	2767/8653	0.78 (0.62–0.98)	**0.04**	34	0.06	0.76		
TC vs. CC	5	14	2797/8714	0.93 (0.80–1.09)	0.38	55	0.55	0.83		
TT+TC vs. CC	5	14	2797/8714	0.90 (0.77–1.06)	0.21	61	0.45	0.74		
TT vs. TC+CC	4	13	2767/8653	0.84 (0.71–1.01)	0.06	7	0.18	0.76		

CI: confidence interval; GA: geographic atrophy; OR: odds ratio; *TLR3*: *Toll-like receptor 3*.

**Table 3:** Meta-analysis of *TLR3* rs3775291 in neovascular age-related macular degeneration.

Genetic model	No. of studies	No. of cohorts	Pooled sample size (nAMD/control)	Test of association			Publication bias (P)			
				OR (95% CI)	P	*I*^*2*^ (%)	Egger's Test	Begg's test		
T vs. C	6	11	2746/3507	0.99 (0.89–1.09)	0.77	29	0.95	0.88		
TT vs. CC	5	10	2664/3446	0.82 (0.66–1.04)	0.10	20	0.90	0.72		
TC vs. CC	6	11	2746/3507	1.08 (0.94–1.25)	0.27	36	0.40	0.64		
TT+TC vs. CC	6	11	2746/3507	1.04 (0.91–1.19)	0.57	35	0.59	0.64		
TT vs. TC+CC	5	10	2664/3446	0.78 (0.64–0.94)	**0.01**	0	0.89	0.59		

CI: confidence interval; nAMD: neovascular age-related macular degeneration; OR: odds ratio; *TLR3*: *Toll-like receptor 3*.
