# Supplementary material for: Association of toll-like receptor 3 polymorphism rs3775291 with age-related macular degeneration: a systematic review and meta-analysis
Source: Sci Rep. 2016 Jan 22;6:19718. doi: 10.1038/srep19718 (PMC4726375; doi:10.1038/srep19718)
Supplement: Supplementary Information [file srep19718-s1.pdf]

Association of *toll-like receptor 3* polymorphism rs3775291 with age-related macular degeneration: a systematic review and meta-analysis

Li Ma,[1] Fang Yao Tang,[1] Wai Kit Chu,[1] Alvin L. Young,[1,2] Marten E. Brelen,[1,2] Chi Pui Pang,[1,2] Li Jia Chen.[1,2*]

[1]Department of Ophthalmology and Visual Sciences, The Chinese University of Hong Kong, Hong Kong, China, [2]Department of Ophthalmology and Visual Sciences, Prince of Wales hospital, Hong Kong, China.

Correspondence:

Dr Li Jia Chen

Department of Ophthalmology & Visual Sciences, The Chinese University of Hong Kong, Hong Kong Eye Hospital, 147K, Argyle Street, Kowloon, Hong Kong.

Tel: +852 39435810 Fax: +852 27159490 E-mail: lijia_chen@cuhk.edu.hk

Table S1. Genetic distributions of *toll-like receptor 3* polymorphism rs3775291 in the included cohorts and estimation of HWE

First author (year)	Ethnicity	All forms of AMD				nAMD				GA				Control				P_{HWE}
		N	TT	TC	CC	N	TT	TC	CC	N	TT	TC	CC	N	TT	TC	CC	
Yang Z. (2008)	Caucasian (Utah 1)	825	51	368	406	441	29	201	211	232	14	93	125	359	40	163	156	0.79
	Caucasian (Utah 2)	450	31	176	243	179	12	81	86	271	19	95	157	421	42	196	183	0.28
	Caucasian (AREDS)	184	4	68	112	NA	NA	NA	NA	184	4	68	112	134	11	62	61	0.39
	Chinese (Chengdu, China)	140	8	56	76	140	8	56	76	NA	NA	NA	NA	171	17	62	92	0.18
Cho Y. (2009)	Caucasian (NEI)	130	12	60	58	NA	NA	NA	NA	NA	NA	NA	NA	187	16	65	106	0.19
	Caucasian (AREDS)	406	24	180	202	NA	NA	NA	NA	125	7	56	62	198	17	89	92	0.24
	Caucasian (BMES)	276	28	115	133	NA	NA	NA	NA	NA	NA	NA	NA	551	63	226	262	0.18
	Caucasian (combined)	465	35	207	223	284	19	132	133	181	16	75	90	936	96	380	460	0.18
Allikmets R. (2009)	Colombian	211	13	93	105	NA	NA	NA	NA	211	13	93	105	365	28	133	204	0.58
	Caucasian (Iowa)	102	12	37	53	NA	NA	NA	NA	102	12	37	53	295	35	108	152	0.09
	Caucasian (Amsterdam)	89	8	40	41	NA	NA	NA	NA	89	8	40	41	264	25	103	136	0.67
	Caucasian (Rotterdam)	64	7	29	28	NA	NA	NA	NA	64	7	29	28	843	80	341	422	0.63
	Caucasian (Würzburg, Germany)	184	16	63	105	NA	NA	NA	NA	184	16	63	105	366	36	139	191	0.33
	Caucasian (Reykjavik, Iceland)	210	12	96	102	NA	NA	NA	NA	210	12	96	102	169	14	65	90	0.93
	Caucasian (AREDS)	163	10	71	82	NA	NA	NA	NA	163	10	71	82	204	21	82	101	0.80
	Caucasian (Melbourne, Australia)	57	6	21	30	NA	NA	NA	NA	57	6	21	30	163	17	76	70	0.82
Edward AO. (2009)	Caucasian (USA)	267	23	129	115	178	17	88	73	89	6	41	42	222	17	81	124	0.46
	Caucasian (USA)	656	59	291	306	377	35	171	171	279	24	120	135	317	35	124	158	0.16
	Caucasian (USA)	864	64	382	418	646	37	304	305	218	27	78	113	479	34	210	235	0.16
	Caucasian (AREDS)	431	38	190	203	247	22	108	117	184	16	82	86	171	17	62	92	0.24
Sng CC. (2011)	Chinese (Singapore)	126	19	63	44	126	19	63	44	NA	NA	NA	NA	274	39	130	105	0.90
Yu Y. (2011)	Caucasian (TMMG)	819	74	344	401	NA	NA	NA	NA	819	74	344	401	4134	348	1702	2084	0.98
Cipriani V. (2012)	Caucasian (UK)	893	78	372	443	NA	NA	NA	NA	NA	NA	NA	NA	2199	180	898	1121	0.99
Cheng Y. (2014)	Chinese (Beijing, China)	46	2	17	27	46	2	17	27	NA	NA	NA	NA	96	8	36	52	0.62
Sharma NK. (2014)	Indian (Chandigarh)	112	6	33	73	82	5	22	55	30	1	11	18	61	0	27	34	**0.026**

AMD: age-related macular degeneration; AREDS: age-related eye disease study; BMES: blue mountains eye study; GA: geography atrophy; HWE: Hardy-Weinberg equilibrium; nAMD: neovascular age-related macular degeneration; N: number of sample size; NA: not available; NEI: national eye institute: TMMG: Tufts/MMAP/MIGen/GAIN (Tufts- Tufts/Massachusetts General Hospital (MGH) GWAS Cohort Study; MMAP-Michigan, Mayo, AREDS, Pennsylvania Cohort Study; MIGen- the Myocardial Infarction Genetics Consortium; GAIN- the Genetic Association Information Network).

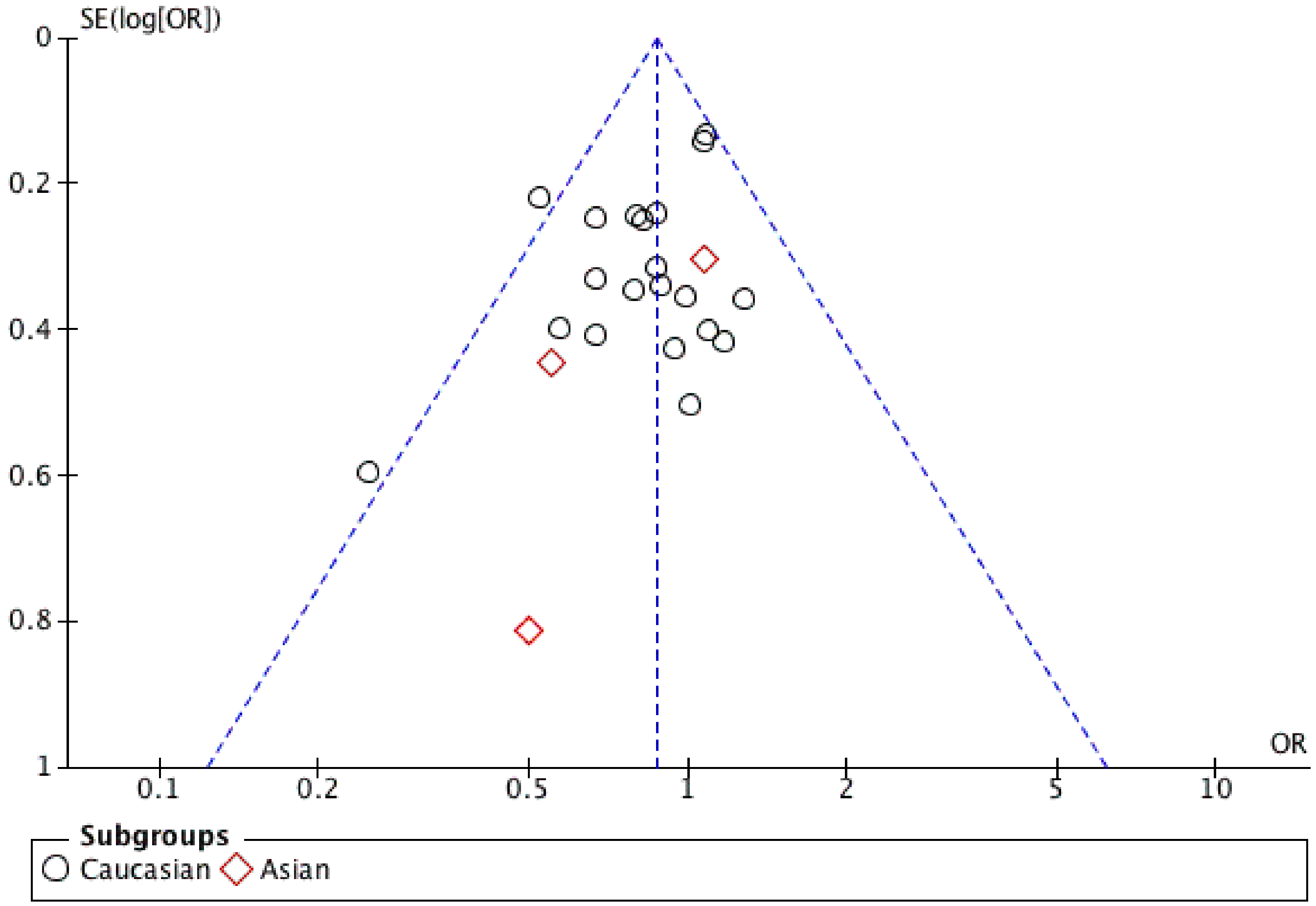

Figure S1. Funnel plot of *TLR3* rs3775291 in the recessive model.

Supplementary Note. Database search

Search strategy for EMBASE

1 AMD.mp.

2 Age-related macular degeneration.mp.

3 ARMD.mp.

4 Age-related macular disease.mp.

5 Age-related maculopathy.mp.

6 ARM.mp.

7 1 or 2 or 3 or 4 or 5 or 6

8 TLR3.mp.

9 toll-like receptor 3.mp.

10 toll like receptor 3.mp.

11 8 or 9 or 10

12 7 and 11

Search strategy for PubMed:

("macular degeneration"[MeSH Terms] OR ("macular"[All Fields] AND "degeneration"[All Fields]) OR "macular degeneration"[All Fields] OR ("age"[All Fields] AND "related"[All Fields] AND "macular"[All Fields] AND "degeneration"[All Fields]) OR "age related macular degeneration"[All Fields]) OR ARMD[All Fields] OR (Age-related[All Fields] AND macular[All Fields] AND ("disease"[MeSH Terms] OR "disease"[All Fields])) OR ("age"[All Fields] AND "related"[All Fields] AND "maculopathy"[All Fields]) OR "age related maculopathy"[All Fields]) OR ("ARM"[MeSH Terms] OR "ARM"[All Fields]) OR

AMD[All Fields]) AND (TLR3[All Fields] OR ("toll-like receptor 3"[MeSH Terms] OR "toll-like receptor 3"[All Fields] OR "toll like receptor 3"[All Fields])

Search strategy for Web of Science:

(AMD or Age-related macular degeneration or ARMD or Age-related macular disease or Age-related maculopathy or ARM) and (TLR3 or toll-like receptor 3 or toll like receptor 3)
